# Utility of Circulating Tumor DNA to Assess Tumor Response in Patients with Locally Advanced Rectal Cancer Undergoing Neoadjuvant Therapy

**DOI:** 10.3390/cancers18040589

**Published:** 2026-02-11

**Authors:** Sakti Chakrabarti, Stacey A. Cohen, Antony Tin, Autumn Dangl, Ki Y. Chung, Mohamedtaki A. Tejani, Marwan G. Fakih, Sreenivasa R. Chandana, Colleen A. Donahue, Virgilio George, Midhun Malla, Vasily N. Aushev, Giby V. George, J. Bryce Ortiz, Whitney K. Herter, Arun Nagarajan, Benjamin A. Weinberg, Vivek R. Sharma, Gregory P. Botta, May Cho, Georges Azzi, Anup Kasi, Farshid Dayyani, Diana L. Hanna, Bradley G. Somer, Meenakshi Malhotra, Shruti Sharma, Adham Jurdi, Minetta C. Liu, Ron G. Landmann, Arvind Dasari

**Affiliations:** 1 University Hospitals Seidman Cancer Center, Case Western Reserve University, Cleveland, OH 44106, USA; 2 Fred Hutchinson Cancer Center, University of Washington School of Medicine, Seattle, WA 98109, USA; shiovitz@uw.edu; 3 Natera, Inc., Austin, TX 78753, USAvaushev@natera.com (V.N.A.); mliu@natera.com (M.C.L.); 4 Prisma Health Cancer Institute, Greenville, SC 29605, USA; 5 AdventHealth, Altamonte Springs, FL 32701, USA; 6 City of Hope Comprehensive Cancer Center, Duarte, CA 91010, USA; mfakih@coh.org; 7 The Cancer & Hematology Centers, Grand Rapids, MI 49503, USA; 8 Department of Surgery, Medical University of South Carolina, Charleston, SC 29425, USAgeorgev@musc.edu (V.G.); 9 Division of Hematology and Oncology, University of Alabama, Birmingham, AL 35294, USA; 10 Cleveland Clinic Florida, Weston, FL 33331, USA; 11 Medstar Georgetown University Hospital, Washington, DC 20007, USA; 12 University of Louisville, Louisville, KY 40202, USA; vivek.sharma@louisville.edu; 13 University of California San Diego Moores Cancer Center, La Jolla, CA 92093, USA; 14 University of California, Irvine, CA 92697, USA; 15 Holy Cross Health-Fort Lauderdale, Fort Lauderdale, FL 33308, USA; 16 University of Kansas Medical Center, Kansas City, KS 66103, USA; 17 Chao Family Comprehensive Cancer Center, University of California Irvine Health, Orange, CA 92868, USA; 18 USC Norris Comprehensive Cancer Center, Los Angeles, CA 90033, USA; 19 West Cancer Center & Research Institute, Memphis, TN 38138, USA; 20 Baptist MD Anderson Cancer Center, Jacksonville, FL 32207, USA; 21 University of Texas, MD Anderson Cancer Center, Houston, TX 77030, USA

**Keywords:** rectal cancer, LARC, TNT, ctDNA, non-operative management

## Abstract

Background: Circulating tumor (ct)DNA is a prognostic biomarker in gastrointestinal malignancies. In rectal cancer, its utility to inform perioperative management and predict recurrence, particularly in patients undergoing non-operative management (NOM), remains unclear. Studies are needed to clarify how post-neoadjuvant therapy (NAT) and post-surgical ctDNA status correlate with clinical outcomes in localized rectal cancer. Methods: We retrospectively analyzed ctDNA data from 220 patients with rectal cancer using a personalized tumor-informed assay (Signatera™, Natera, Inc., Austin, TX, USA). Of these, 148 (67.3%) underwent NAT followed by surgery, and 72 (32.7%) underwent NAT followed by NOM. We assessed associations between post-NAT ctDNA status and survival outcomes. In the surgical cohort, we examined associations between post-operative ctDNA status and clinical response, pathological response, survival outcomes, and NAR scores. Results: In the surgical cohort, ctDNA positivity at the post-operative MRD timepoint was a strong predictor of recurrence, with an 88.3% relapse rate compared to 11.5% in ctDNA-negative patients (*p* < 0.001). Among the 64 NOM patients with post-NAT ctDNA, 21.9% (14/64) were ctDNA-positive, of whom 100% (14/14) relapsed (92.9% local-only), 13 relapsed by the time of data cut-off, and one relapsed 8 months after the cut-off. Only 10% (5/50) of the ctDNA-negative NOM patients experienced local recurrence (*p* < 0.0001). ctDNA positivity post-NAT was associated with inferior DFS (*p* = 0.003). Conclusion: ctDNA was a strong predictor of recurrence in rectal cancer, including in NOM settings. In NOM patients, ctDNA detected local recurrences, highlighting its potential to guide post-NAT surveillance and treatment.

## Simple Summary

Here, we evaluated whether circulating tumor DNA (ctDNA) measured after neoadjuvant therapy (NAT) or surgery can help to assess tumor response and predict recurrence risk in patients with locally advanced rectal cancer, including those managed non-operatively (NOM). In this large real-world study of 220 patients, ctDNA positivity after NAT or surgery was strongly correlated with recurrence risk. Among the NOM patients, post-NAT ctDNA positivity identified individuals at nearly universal risk of local regrowth, while ctDNA negativity predicted durable responses. In the surgical patients, ctDNA clearance after resection was associated with favorable outcomes. ctDNA provides a molecular measure of treatment response that complements radiographic and endoscopic assessment. These findings suggest that ctDNA may help to tailor local therapy and surveillance strategies in rectal cancer and warrant validation in prospective ctDNA-guided trials.

## 1. Introduction

The management of locally advanced rectal cancer (LARC) is guided primarily by clinical stage [1,2,3]. Standard treatment for stages II–III disease includes neoadjuvant therapy (NAT), followed by surgery for those with residual disease, and selective use of adjuvant chemotherapy. For patients achieving a complete clinical response (cCR) after NAT, as assessed by radiographic and endoscopic evaluation, non-operative management (NOM) is considered [3,4,5]. The options for NAT include chemoradiation (CRT) alone or total neoadjuvant therapy (TNT), in which multiagent chemotherapy and CRT are delivered preoperatively, with TNT increasingly favored [6,7]. Phase III trials have demonstrated that TNT reduces disease-related treatment failure (RAPIDO) and improves overall survival (PRODIGE 23) [8,9,10,11,12]. TNT also increases cCR rates (20–40% vs. 10–20% with CRT), expands opportunities for NOM, and reduces distant metastases [1,8,13,14,15].

Approximately 10–30% of patients with localized rectal cancer achieve pathological complete response (pCR) [16,17]. Identifying patients with substantial tumor regression after NAT is critical for selecting candidates for organ-preserving strategies while avoiding post-operative morbidity [18]. However, clinical assessment remains imperfect: cCR does not reliably equate to pCR or absence of molecular residual disease (MRD) [19], and patients managed with NOM continue to face substantial risks of local regrowth or distant recurrence [20,21].

In recent years, circulating tumor DNA (ctDNA) assays have emerged as important investigational tools in localized and metastatic colorectal cancer [22,23,24,25]. In early-stage colon cancer, post-operative ctDNA detection is a strong predictor of recurrence [26,27]. Yet, the relevance of ctDNA detection after NAT or surgery in LARC, and its potential role in NOM, remain undefined.

To address this gap, we analyzed real-world data from patients with LARC treated with standard NAT, surgery, or NOM. Our goal was to evaluate the prognostic potential of ctDNA in LARC, including its ability to identify patients at increased risk of recurrence after NAT or surgery and those undergoing NOM.

## 2. Materials and Methods

### 2.1. Subjects and Study Design

A total of 283 patients with rectal cancer who underwent testing using a tumor-informed ctDNA assay, Signatera™ (Natera, Inc., Austin, TX, USA), from multiple institutions between April 2018 and July 2024 were eligible for analysis. Tests were ordered commercially according to the provider’s clinical practice, and patients (stages II–III) meeting the inclusion criteria (*n* = 220, 1572 plasma samples) were identified retrospectively. Criteria for patient inclusion were confirmed stages II–III LARC and receipt of NAT followed by surgical resection or NOM. Post-NAT clinical response used to stratify patients for surgery or for NOM was determined by the ordering physician based on post-NAT MRI, digital rectal exam (DRE), and proctoscopy results.

**Figure 1:**
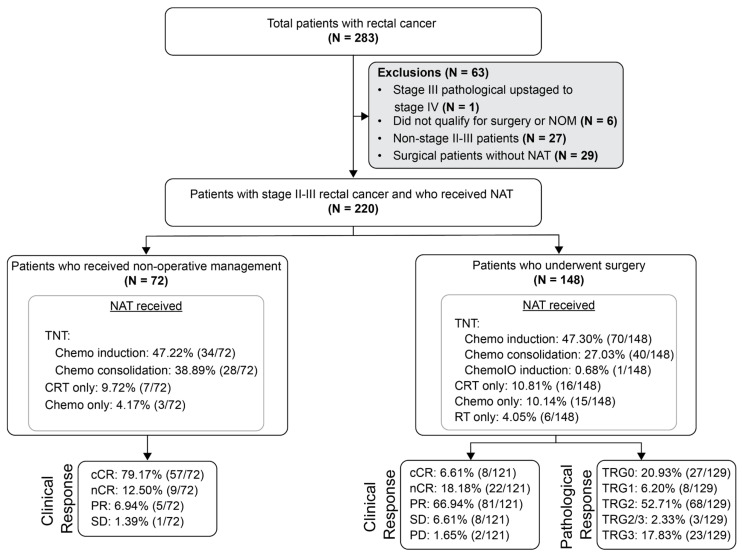
Flow diagram of patient inclusion and exclusion criteria for analysis. NAT: neoadjuvant therapy; RT: radiation therapy; TNT: total neoadjuvant therapy; CRT: chemoradiation; cCR: clinical complete response; nCR: near-complete response; PR: partial response; SD: stable disease; PD: progressive disease; TRG: tumor regression grade.

Patients were assigned to the surgical cohort if they underwent surgery following NAT. For patients in the surgical cohort, the MRD window was defined as 2–12 weeks after surgery prior to the initiation of NAT. Patients were assigned to the NOM cohort if, following NAT, they were initially managed without immediate surgery regardless of whether salvage TME surgery was later performed for local regrowth. Patients achieving complete clinical response (cCR) or near-complete response (nCR) were considered eligible for NOM. A small number of patients with either partial response (PR) or stable disease (SD) were included in the NOM cohort at the provider’s discretion or due to patient choice. Patients who underwent upfront surgery without NAT (N = 29); those who were not eligible for surgery or NOM, declined surgery, or were inoperable despite having gross residual disease post-NAT (N = 6); or who were not stages II–III (N = 27) were excluded (Figure 1).

This study was conducted in compliance with Natera’s Institutional Review Board (IRB)-approved protocol (Salus #21204-02B), the Declaration of Helsinki, Title 21 of the US Code of Federal Regulations (CFR) as applicable, Good Clinical Practice guidelines, and International Conference on Harmonization guidelines. A waiver of the consent process and of the requirement for documentation of informed consent was granted according to 45 CFR 46.116(d) and 45 CFR 46.117(c)(2), respectively.

### 2.2. Personalized ctDNA Assay Workflow

For all patients, blood specimens (two 10 mL Streck tubes) were collected at the discretion of the ordering physician, with an average of 7 samples per patient (range: 1–28). All biological specimens were processed according to a CLIA-validated standard operating procedure at Natera, Inc. ctDNA analysis was performed using a clinically validated, personalized, and tumor-informed 16-plex PCR next-generation sequencing (NGS) assay (Signatera™, Natera, Inc., Austin, TX, USA) in patients undergoing commercial ordering as previously described [28]. Briefly, whole-exome sequencing (WES) was performed on formalin-fixed and paraffin-embedded tumor tissue, along with matched normal DNA blood samples from each patient. Based on the WES results, a personalized assay consisting of multiplex PCR (mPCR) primers was designed for 16 high-ranked patient-specific somatic single-nucleotide variants (SNVs) for each patient. The mPCR assay was then utilized in the associated patients’ plasma-derived cfDNA to detect and track ctDNA. Plasma cfDNA samples with ≥2 SNVs above a predefined algorithmic confidence threshold were considered ctDNA-positive. ctDNA concentration (levels) was quantitatively reported as mean tumor molecules (MTMs) per mL of plasma.

### 2.3. Statistical Analyses

For surgical patients, the primary outcome was disease-free survival (DFS), defined as the time from surgery to endoscopic or radiological recurrence (locoregional or distant) or death, and censored at last follow-up or death. For NOM patients, the primary outcome was TME-free survival, measured as time from NAT completion to salvage surgery or death. Survival analysis was performed using the Kaplan–Meier method and R software (v4.3.1; RRID:SCR_000432). To account for immortal time bias, we applied a landmark analysis, restricting post-operative MRD assessment to the 2–12-week window after surgery and only including patients who were alive and event-free until ≥12 weeks after surgery. A multivariable Cox proportional hazards model was used to assess the most significant prognostic factor associated with DFS. To account for variability in the timing and frequency of ctDNA sampling during surveillance, ctDNA status was modeled as a time-varying covariate in a time-dependent Cox regression for some analyses. All *p*-values were based on two-sided testing and considered significant at *p* ≤ 0.05. Time-dependent Cox regression evaluated serial ctDNA analysis during surveillance.

## 3. Results

### 3.1. Patient Cohort

A total of 1572 plasma samples were collected from 220 patients with stages II–III rectal cancer (median age: 59 years [range: 32–89 years]). Specific details regarding tumor type, pathologic stage, and treatment are described in Table 1. In total, 32.9% (72/220) received non-operative management (NOM) after NAT, and 67.12% (148/220) of the patients underwent surgery following NAT (Figure 1). NAT included total neoadjuvant therapy (TNT), chemotherapy alone, and chemoradiation/radiation alone.

For the surgical cohort, ctDNA at the post-NAT timepoint was collected at a median of 1.4 months (range: 0.5–6.3 months) after the completion of planned NAT, prior to surgery, with a median length of time between completion of NAT and surgery of 68 days (range: 6–270 days). For the NOM cohort, the first timepoint collected was at a median of 2.9 months (range: 0–40 months) after completion of planned NAT. All serial post-NAT ctDNA timepoints from the completion of NAT up to the time of recurrence or last follow-up, if no relapse, were evaluated. In the NOM cohort, 32 patients had their first available ctDNA within 3 months post-NAT; 6.3% (2/32) were ctDNA-positive, both of whom experienced local recurrence. Of the remaining 30 ctDNA-negative patients, 20% (6/30) converted to ctDNA-positive during serial testing, and all subsequently developed local recurrence, and none developed distant metastatic recurrence.

### 3.2. NOM Cohort: Post-NAT Association of Clinical Response and ctDNA Status with DFS

**Figure 2:**
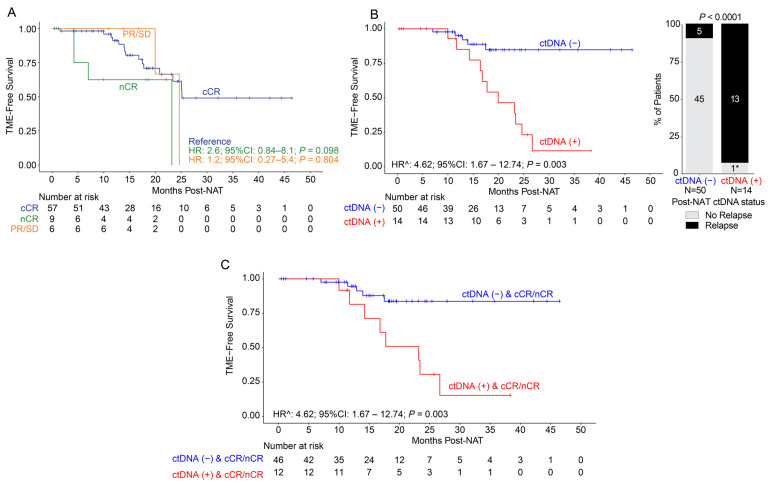
ctDNA and clinical response of patients with NOM management. (**A**) Kaplan–Meier estimates of patients who underwent non-operative management, representing TME-free survival stratified by clinical response post-NAT. (**B**) Kaplan–Meier estimates of TME-free survival stratified by ctDNA status (positive or negative) post-NAT (left panel) and the association of post-NAT ctDNA status with relapse (right panel). (**C**) Kaplan–Meier estimates of TME-free survival stratified by ctDNA status (positive or negative) post-NAT and clinical response (grouped by cCR/nCR only). ctDNA: circulating tumor DNA; CI: confidence interval; HR: hazard ratio; NAT: neoadjuvant therapy; cCR: complete clinical response; nCR: near-complete clinical response; SD: stable disease. * The remaining one patient who did not relapse by the time of data cut-off eventually relapsed 8 months after the end of the data cut-off. ^ Indicates time-dependent hazard ratio.

Of the 72 patients pursuing NOM after NAT, 79.1% (57/72) showed cCR, 12.5% (9/72) showed near-complete response (nCR), 6.9% (5/72) showed partial response (PR), and 1.4% (1/72) showed stable disease (SD). Of the six patients with PR or SD, three eventually achieved cCR without additional treatment intervention. The median follow-up post-NAT was 17 months (range: 1–48). When evaluating TME-free survival by clinical response, no statistically significant differences were observed between responses (Figure 2A).

By ctDNA status, 92.0% (46/50) of the ctDNA-negative patients showed cCR/nCR compared to 85.7% (12/14) of ctDNA-positive patients. Regardless of post-NAT ctDNA status, the clinical response status (cCR/nCR vs. PR/SD) between the ctDNA-negative or positive group was not statistically different, *p* = 1. However, when comparing relapse to non-relapse patients between the post-NAT ctDNA-negative and -positive groups, the difference was statistically significant, *p* < 0.0001 (Figure 2B), suggesting that, in NOM-eligible patients, post-NAT ctDNA can identify the patients who will eventually relapse.

On evaluating TME-free survival stratified by ctDNA status, the ctDNA-positive patients demonstrated significantly inferior outcomes compared to the ctDNA-negative patients (time-dependent HR: 4.62, 95% CI: 1.67–12.74, *p* = 0.003; Figure 2B). Due to the nature of serial testing and variable frequency of plasma collection for monitoring, the post-NAT analysis included ctDNA testing from the completion of NAT up to the time of recurrence or until the last clinical follow-up for patients who did not recur. Among the NOM patients with ctDNA available after completion of NAT (N = 64), 78.1% (50/64) were ctDNA-negative and 21.9% (14/64) were ctDNA-positive. Furthermore, 92.9% (13/14) of the ctDNA-positive post-NAT patients relapsed or progressed compared to only 10% (5/50) of the ctDNA-negative patients. The one remaining patient with ctDNA positivity post-NAT had multiple ctDNA-positive samples accompanied by a suspicious imaging 1 month after turning ctDNA-positive. This patient ultimately relapsed 8 months after the data cut-off. Among the five post-NAT ctDNA-negative patients who relapsed, one patient only had one post-NAT ctDNA draw that was taken 310 days prior to relapse, and another eventually experienced ctDNA positivity immediately after clinical recurrence 154 days later. We observed that the median time to molecular recurrence post-NAT was 8.9 months (range: 0.32–27.8) and the median time to clinical recurrence post-NAT was 13.5 months (range: 8.1–25.1) post-NAT.

Of note, among the 13 post-NAT ctDNA-positive patients who relapsed, all 13 (100%) had local regrowth or local recurrence, except one had both local and lung recurrence. Interestingly, when utilizing clinical response as an adjunct to ctDNA status post-NAT, among the patients who achieved cCR/nCR, ctDNA-positive status remained significantly associated with inferior TME-free survival (time-dependent HR: 4.62, 95% CI: 1.67–12.74, *p* = 0.003; Figure 2C).

### 3.3. Surgical Cohort: Association of ctDNA Status with Pathological Response and DFS

**Figure 3:**
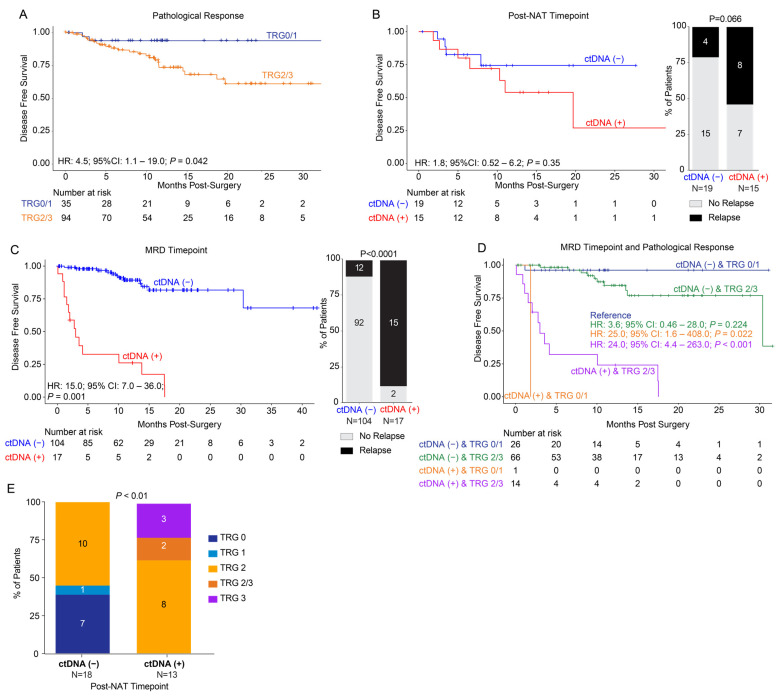
Pathological and ctDNA status response in the surgical cohort post-NAT. (**A**) Kaplan–Meier estimates of patients who underwent surgical resection representing disease-free survival stratified by pathological response post-surgery (grouped by TRG0/1 and TRG2/3). (**B**) Kaplan–Meier estimates of disease-free survival at the post-NAT timepoint stratified by ctDNA status post-NAT, pre-surgery (positive or negative; left panel). Association of ctDNA status at the post-NAT timepoint with relapse status (right panel). (**C**) Kaplan–Meier estimates of disease-free survival at the MRD timepoint stratified by ctDNA status post-surgery (positive or negative; left panel). Association of ctDNA status at the MRD timepoint with relapse status (right panel). (**D**) Kaplan–Meier estimates of DFS stratified by ctDNA at the MRD timepoint in conjunction with pathological response. (**E**) Association of TRG scores with ctDNA negativity and ctDNA positivity at the post-NAT timepoint. ctDNA: circulating tumor DNA; CI: confidence interval; HR: hazard ratio; NAT: neoadjuvant therapy; TRG: tumor regression grade.

A total of 148 patients underwent surgical resection, and, of these, pathological response to neoadjuvant therapy was available for 129 patients. The median clinical follow-up post-surgery was 11 months (range: 0–44). For patients with pathological response data available, 20.9% (27/129) had a TRG 0 score, 6.2% (8/129) were TRG 1, 52.7% (68/129) were TRG 2, 2.3% (3/129) were TRG 2/3, and 17.8% (23/129) were TRG3. Upon evaluating DFS stratified by pathological response, patients with TRG scores 2–3 showed inferior outcomes when compared to patients with TRG scores 0–1 (pathological response: HR: 4.5, 95% CI: 1.1–19.0, *p* = 0.042; Figure 3A).

On stratifying patients with available ctDNA (N = 34) at the post-NAT timepoint, patients with ctDNA positivity showed worse DFS compared to ctDNA-negative patients (HR: 1.8, 95% CI: 0.52–6.2, *p* = 0.35), with relapse rates of 53% (8/15) and 21% (4/19) for the ctDNA-positive and -negative groups, respectively. However, this finding was not statistically significant (Figure 3B). The median follow-up for these 34 patients was 11 months (range: 0–27). Of note, at the post-operative MRD timepoint (N = 121), patients with ctDNA positivity showed a significantly inferior DFS (HR: 15.0; 95% CI: 7.0–36.0, *p* = 0.001. Figure 3C), with a relapse rate of 11.5% (12/104) for ctDNA-negative patients and 88% (15/17) for ctDNA-positive patients (*p* < 0.0001). Of the surgical patients who were ctDNA-positive at the post-NAT timepoint (n = 15), 80% (12/15) also had available ctDNA timepoints during the MRD window. Of these, three were ctDNA-positive in the MRD window, while nine were ctDNA-negative. All three patients with ctDNA positivity had distant recurrences. Of the nine patients who were ctDNA-negative, six remained relapse-free and three eventually relapsed (two distant and one local), with two of these turning ctDNA-positive prior to recurrence during surveillance.

Furthermore, upon assessing DFS based on ctDNA status in conjunction with pathological response, ctDNA-negative patients showed improved DFS regardless of TRG score. By contrast, ctDNA-positive patients with a TRG score of 0/1 (HR: 25.0; 95% CI: 1.6–408.0; *p* = 0.02) or a TRG score of 2/3 (HR: 24.0; 95% CI: 4.4–263.0, *p* < 0.001) displayed significantly inferior DFS (Figure 3D). Additionally, at the post-NAT timepoint, none of the ctDNA-negative patients showed TRG scores 2/3-3, while, among the ctDNA-positive patients, none had a TRG score 0/1. Each category of TRG score was statistically significantly different between the ctDNA-positive and -negative groups (*p* < 0.01; Figure 3E).

### 3.4. Surgical Cohort: NAR Score and Association with ctDNA Status and Outcomes

**Figure 4:**
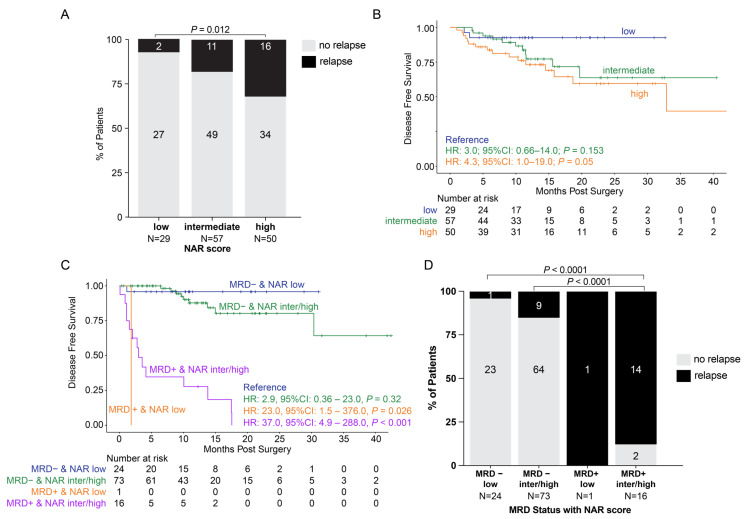
NAR score and association with ctDNA status and outcomes in the NOM and surgical cohorts. (**A**) Association of NAR scores with relapse rates. (**B**) Kaplan–Meier estimates of patients in both the NOM and surgical cohorts representing disease-free survival stratified by NAR score. (**C**) Kaplan–Meier estimates of disease-free survival stratified by ctDNA status at the MRD timepoint (MRD positive or MRD negative) and NAR score (grouped by NAR low, NAR intermediate/high). (**D**) Association of ctDNA status at the MRD timepoint (MRD positive or MRD negative) and NAR score (grouped by NAR low, NAR intermediate/high) with relapse rates. CI: confidence interval; HR: hazard ratio; MRD: molecular residual disease; NAR: neoadjuvant rectal.

In our study, we observed that patients with high NAR scores exhibited a significantly higher recurrence rate of 32% (16/50) when compared to patients with low NAR scores (7% [2/29], *p* = 0.012. Figure 4A). Additionally, we observed that patients with high (HR: 4.3, 95% CI: 1.0–19.0, *p* = 0.05) and intermediate (HR: 3.0, 95% CI: 0.66–14.0, *p* = 0.153) NAR scores exhibited inferior DFS when compared to patients with low NAR scores (Figure 4B). When assessing ctDNA status at the post-op MRD timepoint in conjunction with the NAR score, we found that patients with high NAR scores were more frequently ctDNA-positive (27% [14/51]) than patients with intermediate (11% [6/54], *p* = 0.05) and low (4% [1/25], *p* = 0.02) NAR scores.

When evaluating DFS, we found that the use of ctDNA status at the MRD window enhances the predictive utility of the NAR score. While patients with intermediate or high NAR scores had a worse prognosis than those with low NAR scores, the majority of intermediate and high NAR scores did not recur (81% and 68%, respectively). When coupled with ctDNA status at the MRD window, ctDNA can further stratify the NAR low, intermediate, and high patients and predict relapse. As shown in Figure 4C, regardless of NAR score, patients who were ctDNA-positive in the MRD window had inferior outcomes compared to patients who were ctDNA-negative (ctDNA-positive with low NAR: HR: 23.0, 95% CI: 1.5–376.0, *p* = 0.026; ctDNA-positive with intermediate/high NAR: HR: 37.0, 95% CI: 4.9–288.0, *p* < 0.001). There was no statistically significant difference between ctDNA-negative patients with low and intermediate/high NAR scores. Finally, the relapse rates were higher in patients who were ctDNA-positive when compared to patients who were ctDNA-negative regardless of NAR score (*p* < 0.0001 for all comparisons. Figure 4D).

## 4. Discussion

ctDNA has emerged as a prognostic biomarker and shows the potential to transform response assessment and recurrence risk stratification in patients with LARC, particularly as treatment paradigms shift toward organ preservation. In this largest-to-date real-world study of ctDNA in LARC, we demonstrate the clinical relevance of ctDNA testing at both post-neoadjuvant and post-surgical timepoints across patients managed with surgery or NOM. Our findings reveal that ctDNA positivity robustly predicts recurrence, even among patients with radiographic or endoscopic complete response, highlighting its value in informing critical treatment decisions.

Importantly, this study offers real-world insights on the utility of ctDNA in the management of LARC. Among the surgical patients, particularly those achieving cCR, post-NAT ctDNA negativity may identify candidates for treatment de-escalation and less intensive surveillance. Conversely, in the NOM patients, post-NAT ctDNA positivity was strongly associated with local regrowth, suggesting a role for treatment escalation or earlier salvage surgery. Serial ctDNA testing during surveillance may refine risk stratification, distinguishing durable responders from patients at increased risk of recurrence. Additionally, ctDNA negativity may help to identify patients who are suitable for less intensive surveillance, while ctDNA positivity may inform risk stratification and consideration of closer monitoring or alternative management strategies. However, prospective interventional trials are underway and will be required before ctDNA-guided treatment escalation or de-escalation can be adopted in routine clinical practice. Collectively, these findings support the role of ctDNA as a complementary tool to imaging and endoscopy and provide a rationale for prospective trials evaluating ctDNA-guided management strategies in LARC.

A significant proportion of patients in the current study underwent NOM. Our study provides critical insight into the significance of post-NAT ctDNA status in this group. NOM, first popularized by Habr-Gama et al., is increasingly used for select patients with LARC [5]. A previous study reported baseline ctDNA combined with positive MRI extramural venous invasion to predict response [29]. Here, we found that post-NAT ctDNA status was strongly prognostic of outcomes among NOM patients (time-dependent HR: 4.62, 95% CI: 1.67–12.74, *p* = 0.003) independent of clinical response (cCR/nCR *p* = 0.003). Post-NAT ctDNA negativity was associated with remaining relapse-free, whereas ctDNA-positive patients had a high recurrence rate (13/14, 92.9%), suggesting that these patients may not be suitable for a non-operative approach. It is important to note that the one (7.1%; 1/14) remaining patient who was post-NAT ctDNA-positive recurred 8 months after the data cut-off. Therefore, even among patients with cCR/nCR, those who were ctDNA-positive had a 100% recurrence rate (PPV). Notably, all the ctDNA-positive patients had local recurrences, including the patient who recurred after data lock, with one patient having an additional lung recurrence.

Among the patients in the surgical cohort, we found that post-NAT ctDNA status correlated with pathological response, suggesting that it may serve as a surrogate for pathological response. An earlier phase 2 randomized trial reported improved 3-year DFS (71.6%) with adjuvant FOLFOX among patients with post-operative stages II–III disease and high-risk features following preoperative chemoradiotherapy [30]. Since then, several studies have demonstrated ctDNA positivity post-NAT to be associated with an increased risk of recurrence among patients with rectal cancer [25,31]. Here, we found that the prognostic performance of ctDNA differed depending on the timing of assessment in surgical patients. While ctDNA positivity at the post-NAT pre-surgical timepoint was associated with inferior DFS, this association did not reach statistical significance, likely reflecting the residual tumor heterogeneity prior to resection, variable timing of sampling, and limited sample size at this timepoint. In contrast, ctDNA status during the post-operative MRD window was a highly robust and statistically significant predictor of recurrence, consistent with the concept that definitive surgical clearance establishes the most informative biological context for MRD assessment. These findings suggest that ctDNA testing after surgical resection represents a reliable prognostic window for recurrence risk stratification in surgically managed patients with LARC.

Consistent with the published data [25,31,32,33], we found ctDNA positivity during the MRD window (2–12 weeks post-surgery) to be associated with poor outcomes (HR: 15.0, 95% CI: 7.0–36.0, *p* = 0.001). As previously described, the neoadjuvant rectal (NAR) score, calculated using the clinical tumor stage and pathological nodal and tumor stage, can be used as a composite short-term assessment of NAT response [34,35]. We observed that MRD ctDNA status was associated with NAR score, thereby substantiating our previous findings. When used as an adjunct to the NAR score, MRD ctDNA status was found to be the main driver of DFS when compared to the NAR score alone. These patients may benefit from ctDNA-specific clinical trials to address biochemical recurrence. Finally, it is worth noting that local relapse was more frequent among the NOM patients, whereas distant relapse predominated in the surgical cohort.

Our study has several limitations inherent to its real-world retrospective design. The treatment approaches varied across sites, including differences in NAT regimens and NOM implementation. ctDNA testing was performed at the discretion of the treating physicians, resulting in variability in timing and frequency of sampling. This may introduce selection bias as testing may have been preferentially performed in patients perceived to be at higher risk or with equivocal clinical responses. Moreover, while this heterogeneity may introduce confounding, it also reflects contemporary clinical practice and enhances the generalizability of our findings. In some instances, ctDNA results may have influenced clinical decisions in real time, which we acknowledge could affect outcome associations; however, this feature underscores the relevance of ctDNA in routine practice rather than diminishing its potential utility. The follow-up duration was relatively short, which may explain the predominance of local, rather than distant, recurrences observed. An additional limitation relates to the timing of post-NAT ctDNA assessments. Since this was not a prospectively designed study, only a small subset of patients underwent ctDNA testing at standardized timepoints post-NAT, precluding statistically meaningful subgroup analyses for clinical decision-making at the time of NOM consideration. Additionally, the median follow-up after surgery was relatively short, which may preferentially capture early local recurrences and underestimate the incidence of later distant metastases. Longer follow-up will be important to fully define the temporal relationship between the ctDNA dynamics and patterns of recurrence in this population. Despite these limitations, our study results are hypothesis-generating and represent the largest-to-date real-world cohort of patients with LARC undergoing NOM or surgery with serial ctDNA testing. The consistent associations we observed across multiple analyses support the robustness of our findings and highlight the potential of ctDNA to inform both surveillance and treatment decision-making.

## 5. Conclusions

In this large real-world cohort of patients with LARC, ctDNA status at both post-NAT and MRD timepoints correlated with clinical response, pathological response, and recurrence outcomes. ctDNA testing may offer significant value for real-time clinical decision-making, particularly in selecting patients for NOM or intensified post-operative surveillance. Future prospective studies are warranted to validate these findings and to evaluate ctDNA-guided management strategies in clinical trials.

## Figures and Tables

**Table 1:** Cohort demographics, clinicopathologic features, and treatment lines.

Characteristics	N = 220	%
Gender		
Male	123	55.9%
Female	97	44.1%
Median Age (range)	59 (32–89)	
Cohort		
NOM	72	32.7%
Surgical	148	67.3%
Clinical Stage		
II	53	24.1%
NOM	21	9.5%
Surgical	32	14.5%
III	167	75.9%
NOM	51	23.2%
Surgical	116	52.7%
Pathological Stage (surgical cohort)		
0	24	16.2%
I	29	19.6%
II	34	23.0%
III	52	35.1%
Unknown	9	6.1%
Clinical → Pathological Staging (surgical cohort)		
Upstaged	3	2.0%
Downstaged	76	51.4%
Unchanged	60	40.5%
N/A	9	6.1%
MSI Status		
MSS	217	98.6%
MSI	3	1.4%
Vital Status		
Alive	217	99.1%
Deceased	3	0.9%
Median NAR Score	15	-
Clinical Efficacy (months)		
Median DFS	11	-
Median TME-free survival	15	-
Median follow-up	24	-
Pathological Response to NAT		
TRG 0	27	12.3%
TRG 1	8	3.6%
TRG 2	68	30.1%
TRG 2/3	3	1.4%
TRG 3	23	10.5%
N/A	19	8.6%
Clinical Response to NAT		
cCR	63	28.6%
NOM	57	25.9%
Surgical	8	3.6%
nCR	31	14.1%
NOM	9	4.1%
Surgical	22	10.0%
PR	86	39.1%
NOM	5	2.3%
Surgical	81	36.8%
SD	9	4.1%
NOM	1	0.5%
Surgical	8	3.6%
PD	2	0.9%
NOM	0	-
Surgical	2	0.9%
Unknown	28	12.7%
NOM	1	0.5%
Surgical	27	12.3%
Neoadjuvant Treatment Regimen		
TNT	198	90.0%
Chemo Induction	104	47.3%
NOM	34	15.5%
Surgical	70	31.8%
Chemo Consolidation	68	30.9%
NOM	28	12.7%
Surgical	40	18.2%
Chemoradiation Only	23	10.5%
NOM	7	3.2%
Surgical	16	7.3%
ChemoIO Induction	1	0.5%
NOM	0	-
Surgical	1	0.5%
Chemotherapy	18	8.2%
NOM	3	1.4%
Surgical	15	6.8%
Radiotherapy	6	2.7%
NOM	0	-
Surgical	6	2.7%
Recurrence Site		
NOM	24	-
Local	22	92%
Lung + local	2	8%
Surgical	34	-
Lung	13	38%
Local	5	15%
Peritoneum	5	15%
Liver	3	9%
Pelvis	3	9%
Liver + bone	1	3%
Liver + lung	1	3%
Lung + local	1	3%
Lung + lymph node	1	3%
Vagina	1	3%

## Data Availability

The authors declare that all relevant non-proprietary data used to conduct the analyses are available within the article. To protect the privacy and confidentiality of the patients in this study, clinical data are not made publicly available in a repository but can be requested at any time from the corresponding author. All data shared will be de-identified.

## Abbreviations

The following abbreviations are used in this manuscript:

ctDNA	Circulating tumor DNA
MRD	Molecular residual disease
LARC	Locally advanced rectal cancer
TME	Total mesorectal excision
NOM	Non-operative management
NAR	Neoadjuvant rectal (score)
NAT	Neoadjuvant therapy
TNT	Total neoadjuvant therapy
CRT	Chemoradiation
TRG	Tumor regression grade
pCR	Pathological complete response
cCR	Complete clinical response
nCR	Near complete response
PR	Partial response
SD	Stable disease
PD	Progressive disease
DRE	Digital rectal exam
MTM	Mean tumor molecules
WES	Whole exome sequencing
NGS	Next generation sequencing
mPCR	Multiplex polymerase chain reaction
SNV	Single nucleotide variants
DFS	Disease-free survival
HR	Hazard ratio
CI	Confidence Interval
